# Oxidative Stress and Cancer Risk in Schistosomiasis

**DOI:** 10.1155/omcl/9701021

**Published:** 2024-12-17

**Authors:** Justice Afrifa, Eric Gyamerah Ofori, Yeboah Kwaku Opoku, Kwame Kumi Asare, Rosemary Doe Sorkpor, Ibrahim W. Naveh-Fio, Richard Armah, Sandra Ofori, Richard K. D. Ephraim

**Affiliations:** ^1^Department of Medical Laboratory Science, School of Allied Health Sciences, University of Cape Coast, Cape Coast, Ghana; ^2^Department of Biology Education, Faculty of Science Education, University of Education, Winneba, Ghana; ^3^Infectious and Non-Communicable Diseases, Biomedical and Clinical Research Centre, University of Cape Coast, Cape Coast, Ghana; ^4^Inspectorate Directorate, Food and Drugs Authority, Cape Coast P.O. Box CC13733, Ghana

**Keywords:** bladder cancer, oxidative stress, *Schistosoma haematobium*, *Schistosoma mansoni*

## Abstract

**Background:** Schistosomiasis is considered one of the most devastating parasitic diseases globally, coming second only to malaria in terms of morbidity. The disease-causing parasite can inhabit the body for over a decade, leading to imbalances in the host's metabolic systems. The flukes and their eggs can illicit various immunological and metabolic complications resulting in the generation of reactive oxygen species (ROS). These are known to have several devastating effects on the host through increased oxidative stress, DNA mutation, and gene modifications, which can lead to fibrosis and cancer.

**Main Body:** Here, we discuss oxidative stress and cancer risk in Schistosoma infection. The concept of ROS generation and the complex antioxidant systems that enable the parasite to evade oxidant insults and prolong its life span in the host are explored. Further, the various roles of ROS during the initiation and progression of schistosomiasis and its influence on the host are discussed. Finally, mechanisms linked to the risk of bladder cancer in *Schistosoma haematobium* (*S. haematobium*) infections are elucidated.

**Conclusion:** Finally, we provide an opinion on how some of these mechanisms could give directions for future studies as well as provide a springboard for diagnostics and drug targeting in schistosomiasis.

## 1. Introduction

Schistosomiasis, also known as bilharzia, is a tropical parasitic disease caused by trematodes of the genus *Schistosoma*. It is mainly transmitted through a cycle that involves the contamination of surface water with excreta while employing specific freshwater snails as the intermediate host. Schistosomiasis contributes enormously to the global disease burden considering that it is prevalent in some 74 countries and affects over 200 million people worldwide [1–3]. Further, it is estimated that at least 700 million people are at risk of Schistosoma infection, with an estimated worldwide annual mortality rate of around 20,000 [3–5]. A variety of schistosomiasis causative worms exist; however, among humans, *Schistosoma mansoni* (*S. mansoni*), *Schistosoma haematobium*, and *Schistosoma japonicum* (*S. japonicum*) are the major schistosomiasis causing species. *Schistosoma haematobium*, which causes urinary schistosomiasis, is more prevalent in Africa and the Arabian Peninsula and is transmitted by an intermediate host, *Bulinus Snail*. *Schistosoma mansoni*, which is more associated with the liver and intestines is transmitted by *Biomphalaria* snails, and it is commonly found in Africa, the Arabian Peninsula, and South America. The intestinal and hepatosplenic schistosomiasis widely seen in the Philippines, Indonesia, and China could be caused by *S. japonicum* transmitted by the amphibian snail, *Oncomelania* [6]. The economic importance of the burden of *Schistosoma* infections is based on the ability of the disease state to degenerate into serious complications such as colorectal and liver cancers, as has been previously observed in the *S*. *mansoni* and *S*. *japonicum* species [7, 8]. However, the most crucial association has been the link between *S. haematobium* and bladder cancer. Consequently, both the WHO and IARC have classified *S. haematobium* as a Class 1 carcinogen [9]. Schistosomiasis is characterized by over-dispersed population distribution, such that in most endemic areas, children between 5 and 15 years constitute the most significant proportion associated with high infection intensities [10].

The matured schistosome flukes colonize the blood vessels of humans for years with unique abilities to evade the immune system. They rely on blood components such as globulins, plasma proteins, and red blood cells to meet their nutritional needs [6]. During these periods, the worms lay thousands of eggs daily, which could either be trapped in adjacent tissues or be excreted through feces or urine. Several local and systemic pathological conditions are triggered based on the specific immune-mediated granulomatous responses elicited by tissue-trapped eggs. This effect may range from urogenital inflammation (as in the case of *haematobium*), anemia, stunted growth in children, reduced physical fitness, cognitive impairment, periportal fibrosis (PPF), hepatosplenism, and portal hypertension [11]. In some cases, immune resistance could be slowly acquired through a set of complex immune mechanisms [6]. It has long been argued that the parasite's ability to modulate the host's immune response depends on the deposition of eggs. Severe tissue damage and inflammation can result from strong immunological reactions triggered by the release of eggs into host tissues. However, new data supports the idea that all stages of the infection could contribute to immune modulation [12]. Migrating schistosomula, larval, adult worms, and the skin-penetrating cercariae can release biomolecules that modulate both the innate and adaptive immune responses to facilitate parasitic evasion of the host's immune system. This immune modulation may serve dual purposes. On one hand, the attenuated immune responses enhance parasite survival in the host, while at the same time limiting severe host reactions through the modulation of critical immunopathology [13]. Thus, various mechanisms such as the down or upregulation of inflammation or inhibition of cytokines production, coupled with Type 1 helper (Th1) and Type 2 helper (Th2) immune response switches are employed [14]. During the first phase of the parasitic infection, Type 1 inflammatory immune responses within the plasma and tissues are propagated by interferon-*γ*, IL-1, IL-12, and TNF- *α*. However, as the disease progresses to a chronic phase, antigens by deposited eggs trigger the release of CD4+ Th2 responses [15].

Oxidative stress is a major factor in the pathophysiology of schistosomiasis. Hence, the breakdown of the parasite's defensive mechanisms depends heavily on the antioxidant potential of the host. Certain cells are considered significant sources of reactive oxygen species (ROS) and include eosinophils (E), macrophages (M), and neutrophils (N). Inflammations characterized by the infiltration of these cells serve as a hallmark of urogenital schistosomiasis [16]. A clear association between schistosomiasis and endogenous generation of ROS and reactive nitrogen species (RNS)-mediated chronic inflammation has been established [17]. Further, it is known that inducible nitric oxide synthase (iNOS)-mediated oxidative stress is elevated in infectious diseases in response to inflammation [18]. Additionally, oxidative stress associated with lipid peroxidation and DNA damage could aggravate the disease itself. Evidence suggests that schistosomiasis with accompanying oxidative stress is significantly connected with bladder cancer [9, 19]

Therefore, it is vital to understand the state and role of oxidative stress and how it modifies the pathogenesis of urinary schistosomiasis and its consequences, in particularly cancer.

## 2. Oxidative Stress as an Early Process in Schistosomiasis

The host's immune system produces ROS in the early stages of *Schistosoma* infection as a means of combating the parasites' invasion [12]. This results in oxidative stress, especially in the host liver and spleen tissues where the parasites migrate and start the maturation process. Oxidative stress in these organs could lead to tissue damage and inflammation as well as potential damage to cellular constituents such as proteins, lipids, and DNA [12, 20]. This is associated with an early release of cytokines including TNF-*α*, IL-1*β*, and IFN-*γ* by the host immune system. ROS plays several physiological roles, as they are usually formed as oxygen metabolic byproducts or due to the presence of environmental stressors and xenobiotics. ROS may also alter genetic and epigenetic information [21–23].

To compensate for the oxidative damage caused by the host's immunological response, Schistosoma parasites employ a range of antioxidant enzymes, such as catalase, glutathione peroxidase (GPx), and superoxide dismutase (SOD), to neutralize the ROS and enable the parasites to survive in the host's hostile environment [12, 20]. These enzymes are essential in the early stages of infection when the parasites are vulnerable to the immune system's oxidative attack. Even though high levels of oxidative stress can cause tissue damage, fibrosis, and persistent inflammation. It is possible for the parasites' antioxidant defenses to take charge and evade immune destruction leading to the development of a chronic infection [20]. As far back as 1987, it was known that many parasites, including schistosomiasis, have increased susceptibility to oxidative insults compared to their host. This was seen in either their susceptibility to ROS in vivo, increased reserve antioxidant capacity of the host, or drugs that induce oxidative stress [24].

### 2.1. ROS Production by the Host

Generally, M produce ROS to fight invading pathogens, thus, contributing majorly to the net ROS synthesis in hepatic fibrosis [25–27]. In hepatic granuloma cells, M regulate granulomatous inflammation depending on which effector phenotype is activated [28]. In this regard, the differentially activated M M1 and M2 function differently in regulating the inflammation's initiation, progression, and resolution. For instance, in Schistosomiasis, M1 induces a cytotoxic effect on the schistosomula, reducing the risk of hepatic fibrosis in the host [28]. In contrast, M2 macrophage-rich granulomas are generally induced by antigens secreted by schistosome eggs which trigger the release of CD4+ Th2 responses. This prevents acute mortality but enhances the development of liver fibrosis in the chronic stages [15, 28–30]. Interestingly, the differentiation of M2 macrophage depends on ROS but this is not the case for M1 macrophage [31, 32]. In *S. japonicum* infected mice, ROS generation was extensively observed in the liver and it was further demonstrated that *S. japonicum* eggs antigen stimulates the increase in ROS levels in M through the nicotinamide adenine dinucleotide phosphate (NADPH) oxidase 2 (NOX2) pathway [20].

This is in line with the theory that ROS generation during infection is stimulated by the activation of M and is critical for M2 macrophage differentiation. Again, available evidence indicates that *S. japonicum* antigen-associated ROS will preferentially propagate the differentiation of M2 M [33]. This is also true for *S. mansoni*, as *the* maximum intensity of ROS intermediates was observed in M2 macrophage-rich granulomas near *S. mansoni* eggs [34]. Eosinophil cells are associated with schistosome-induced granulomas derived from superoxide and hydroxyl radicals [35]. However, the consequences of ROS generation in schistosomiasis are largely still unknown.

### 2.2. Antioxidant Systems of Schistosomes

Adult *Schistosoma* worms can persist in the human body, an indication that the parasite is efficient in evading or resisting the host's immune response. Parasites in their early developmental stages (including the schistosomula and cercaria phases) are less exposed to redox insults than adult stages [36, 37]. During the schistosomula stage, the parasites have highly developed adequate protective mechanisms for their survival. These include the sequestration of the glycolipids of host erythrocytes at the tegument surface and the lowering of protein antigenic expression [38, 39]. Further, the parasites can enhance complement C3 degradation [40] and initiate a specific mechanism geared towards antioxidant defense buildup in the course of its development [41–43]. Adult *Schistosoma* worms encounter myriads of redox challenges derived from the digestion of blood, activated immune cells, and self-derived aerobic metabolism [44]. Thus, the parasite can survive over a long period by tolerating and detoxifying ROS in the human host, as seen in *S. mansoni* [44]. Specifically for *S. mansoni*, specialized improvement in its antioxidant defense system led to an increase in its capacity to decompose hydrogen peroxide (H_2_O_2_) in the vertebrate host [44–57]. In the bloodstream of the vertebrate host, adult *S. mansoni* can utilize continuous exposure to molecular oxygen (O_2_) to sustain egg production [48, 49], while maintaining cellular energy through oxidative phosphorylation [50, 51]. In *S. mansoni* parasites, various enzymes that metabolize ROS including glutathione reductase, SOD, cytochrome c peroxidase, and GPx have been identified [41]. In schistosomes, there is a developmental-based regulation of redox activity. This is expressed in the levels of antioxidant enzymes such as GPx, cytosolic SOD (CT-SOD), glutathione S-transferase (GST), and signal peptide-containing SOD (SP-SOD). These are minimally expressed in the schistosomula with a relatively elevated expression during schistosome development in the mammalian host, thus, enhancing the resistance action of parasites in the presence of ROS [58, 59]. Similarly, Mei et al. [52] showed that GPx activity was developmentally regulated by observing elevated enzyme activity levels in the extract of adult flukes compared to the larval stages. One of the most important antioxidant enzymes that protects the parasites against oxidant insults is thioredoxin reductase which modulates the intracellular redox environment due to its reductive effect on thioredoxin [53]. Other specific antioxidants detected in *S. mansoni* include 2-cysperoxiredoxins (Prx) 2 and 3 which enable organic peroxides and H_2_O_2_ decomposition by employing glutathione and thioredoxin as electron donors [54–56]. It is interesting to note that, apart from the developmental stages, schistosomes' redox regulation may also be enhanced by the sexes of adult worms. In *S. mansoni* worms, sexual preferences influence nutrient utilization which could regulate ROS generation and endogenous parasite O_2_ consumption. This is linked to the differential contribution of male and female adult worms to redox biology [45]. The nature of the detoxification between male and female adult *S. mansoni* flukes follows different mechanistic pathways. While the male redox homeostasis mechanism is geared towards reducing the generation rate, females are more geared towards detoxification than repression of ROS generation [57, 60, 61].

There is also an important selenoenzyme thioredoxin glutathione reductase (TGR) that prolongs the survival of schistosomes during redox insults in the mammalian host [62]. TGR is a chimeric flavo-enzyme that naturally occurs through the fusion of a thioredoxin reductase domain with a glutaredoxin domain [63, 64]. It mainly functions in the parasites' ROS detoxification pathway ensuring its survival and presents a unique drug target against the parasite [65]. However, irrespective of these extensive mechanisms, the antioxidant capacity of schistosomes is limited compared to the human host [66]. Apart from the lack of catalase [67], lower activity of schistosome GPx proteins in the presence of hydrogen peroxide has also been observed. Due to this, schistosome worms are more likely to be affected by oxidative insults than the human redox pathways. Hence the parasite's redox pathways are possible targets for drugs [68]. Recent studies have shown that auranofin, a gold-containing compound can significantly lower the burden of *S. mansoni* in mice by inhibiting TGR [69].

## 3. Markers of Oxidative Stress in Schistosoma infection

### 3.1. Lipid Peroxidation in Schistosome Infection

Lipid peroxidation occurs when the lipid component of the liver cell membrane gets damaged by excess ROS produced from the immunological response to Schistosoma infection. Byproducts of lipid peroxidation can damage the liver cells and adjacent tissues by releasing inflammatory molecules and causing cell death. Hepatic stellate cells (HSCs) and fibroblasts are activated by the continued generation of inflammatory cytokines and ROS, which promotes fibrogenesis [12, 70]. Collagen and extracellular matrix constituents are produced leading to PPF and scarring in the liver tissues. More serious liver diseases including portal hypertension and liver failure may eventually result from lipid peroxidation [12]. Studies have shown that treating *S. mansoni* not only lowers infection levels but also improves clinical symptoms of hepatosplenomegaly and progressive PPF [68, 71]. A study by Ewuzie et al. [67] suggests that people with current schistosome infection have a 2.5-fold higher chance of developing PFF than people who are not. Ultimately, this can lead to more severe liver conditions like portal hypertension and liver failure [67].

A study that sought to elucidate the possible contribution of products of lipid peroxidation in the hepatic pathophysiology of *S. mansoni-infected* individuals, revealed a significant elevation of plasma malondialdehyde (MDA) among the patients with schistosomiasis compared to the control group [72]. A positive correlation between plasma MDA levels and two hepatic fibrosis parameters: ultrasonography-graded PPF and serum hyaluronic acid has been reported [72]. An insignificant (10%) elevation in plasma MDA among patients infected with *S. mansoni* compared to the controls was reported in another study, which however employed a rather reduced sample size of 18 [58]. Contrary to the above findings, Eboumbou et al. [59] reported that hepatitis rather than *Schistosoma* infection was more likely to induce a higher level of MDA among co-infected individuals. In animal models, *S. mansoni-infected* mice showed higher levels of MDA, which was reduced after the administration of vitamin E and selenium [73]. Due to the scarcity of data and the inconsistencies in existing data, more studies are required to establish the exact role of MDA in *Schistosoma* infection.

Similar to the formation of MDA, conjugated dienes and lipid hydroperoxides, among other products, are formed during the oxidation of polyunsaturated fatty acids and other lipids by intermediate ROS [74]. The levels of erythrocyte-conjugated dienes were significantly elevated in *S. mansoni* individuals compared to the controls [58]. Further, schistosomiasis has been shown to enhance lipid peroxidation by reducing the activity of lecithin–cholesterol acyltransferase, a plasma enzyme essential for cholesterol esterification and the regulation of cell membrane lipid composition in schistosomiasis-infected patients [75]. In mice, excess lipid peroxide generation due to *S. mansoni* infection caused a reduction in antioxidant capacity leading to liver damage [34].

### 3.2. Markers of Oxidative DNA and Protein Damage in Schistosomiasis

The alterations to DNA molecules resulting from ROS overproduction could occur in diverse ways. These include mutations, modifications of purines and pyrimidine bases, and changes in the DNA sugar backbone. ROS can also produce breakages in either a single- or double-stranded DNA, leading to mutations such as deletions or translocations. Typically, ROS-associated oxidative DNA damage occurs by oxidizing purines and pyrimidines at apurinic/apyrimidinic (abasic) DNA sites [76]. Major ROS modifications of DNA that occur endogenously include 2,6-diamino-4-hydroxy-5-formamidopyrimidine and 8-oxo-7,8-dihydroguanine (8-oxoGua). An 8-hydroxy-7,8-dihydroguanyl radical is generated when a hydroxyl radical is added to the C8 position of a guanine ring. Subsequent oxidation or reduction of the 8-hydroxy-7,8-dihydroguanyl radical produces either an 8-oxoGua or the ring-opened hydroxy-5-formamidopyrimidine (FapyGua) [77, 78]. The frequency and extent of these DNA alterations correspond to the intensity and quality of oxidative stress and other accompanying factors. DNA glycosylases in humans are responsible for repairing damaged DNA caused by oxidative-stress-induced base changes such as the development of 8-oxo-2′-deoxyguanosine (8-oxo-dG). By cleaving the damaged base from the DNA backbone, this enzyme starts a base excision repair to prevent long-term genetic instability [79, 80].

In schistosomiasis, there is an increased deposition of eggs in the subepithelial tissues of infected subjects which causes chronic inflammation. This enhances ROS release and makes the DNA prone to oxidative stress lesions [22, 81]. Antigens released by *S. haematobium* eggs deposited in the bladder walls modulate the levels of TNF-*α*, which mediates inflammation in the mononuclear cells of the peripheral blood. The activation of NF-*κ*B leads to iNOS-mediated overproduction of nitric oxide, an essential precursor to 8-nitroguanine and 8-oxodG production. Ma et al. [82] observed that, in cystitis and bladder cancer patients, *S. haematobium*-mediated inflammation increases the population of mutant stem cells. Activation of F-*κ*B leads to DNA damage and subsequent tumorigenesis mediated by iNOS release [82]. In terms of the significant oxidative stress which results in DNA lesions, 8-oxo-dG formation is the most prominent mainly due to its pernicious nature as it is known to cause an impairment in the CpG island methylation of the promoter region of genes [22]. Salim et al. [17] observed an increased level of 8-hydroxy-2-deoxyguanosine (8-OHdG) in schistosomiasis-associated squamous cell carcinomas compared to nonschistosomal carcinomas. In line with that, the researchers observed a significant association of the 8-OHdG with an elevated expression of 8-oxoguanine-DNA-glycosylase and apurinic/apyrimidinic endonuclease which are known DNA repair genes [17]. Again, others have demonstrated the formation of 8-oxo-dG and 8-nitroguanine in tissues of *S. haematobium-infected* bladder cancer patients and further showed a strong correlation between *S. haematobium* infection and oxidative DNA damage [17, 82]. Thus, it may not be surprising that in 8-nitroguanine-positive schistosomiasis-associated bladder tumor cells, NF-*κ*B was colocalized with an increased expression of iNOS [83].

### 3.3. Antioxidant Levels in Schistosomiasis

Naturally, during parasitic infections, various bioactive compounds counteract the progress of infection either as an antioxidant or through an oxidative insult which evades the parasite's antioxidant system [84]. Antioxidant enzymes play a significant role in reducing the severity and progression of schistosomiasis. Thus, with the recognition of schistosome infection as a state of oxidative stress, antioxidants that combat the myriads of ROS released into the host system are essential for recovery. Interestingly, in schistosomal infected baboons, the use of SOD (specifically *S. mansoni* CT-SOD and *S. mansoni* extracellular SOD) and GPx as vaccine components was able to reduce the number of worms compared to controls that were not vaccinated [85]. Again, the enzymatic antioxidant profile in mice models of schistosomiasis revealed a reduction in antioxidants such as catalase, SOD2, and glutathione levels [86, 87]. Others have shown that lipid peroxidation products and nitric oxide were increased with a concomitant decrease in antioxidants such as vitamin E, glutathione, SOD, and catalase activities in the spleen, kidney, and liver of mice infected with *S. mansoni* [88, 89].

## 4. Oxidative Stress and Cancer Risk in Schistosomiasis

Elevated levels of oxidative DNA damage markers such as 8-OHdG and elevated expression of DNA repair genes, 8-oxoguanine-DNA-glycosylase, and apurinic/apyrimidinic endonuclease have been observed in *S. haematobium* infections [17]. The involvement of ROS in carcinogenesis is mainly explained by two mechanisms. First is the ability of ROS to induce genetic mutations stemming from cellular injury. Second, is the effect of ROS on transcriptional factors and signal transducers. However, various factors such as the stress level and type of ROS involved might direct the specific mechanism it follows [90]. ROS are known to cause mutations in some telomere genes as well as damage to some cell cycle-related and tumor suppressor genes such as p53. Again, ROS is a major contributor to the activation of oncogenes (such as Fos and Jun), transcriptional factor NF-*κ*B, and some protein kinases [91].

*Schistosoma haematobium* infection may cause chronic granulomatous cystitis, a precursor to the formation of squamous metaplasia of transitional epithelium leading to subsequent squamous cell carcinoma [92]. Indeed, there is a high incidence of squamous cell carcinoma Schistosomiasis endemic areas [93]. Specifically, Rambau, Chalya, and Jackson [92] reported that, among patients living in the western part of Tanzania, *Schistosoma* eggs were retrieved from about 44.9% of cases diagnosed with urogenital bladder cancer. Again, they reported that schistosomiasis-associated bladder cancer showed more aggressive behavior, as they quickly invaded the muscularis propria of the bladder [92].

Similarly, in the Sokoto region of northern Nigeria, a strong association of chronic schistosomiasis with bladder cancer was observed [94]. Others have shown that, in endemic areas, effective schistosomiasis treatment led to an attenuated occurrence of squamous cell carcinoma [87, 95]. The specific involvement of *S. haematobium* in squamous cell carcinoma could be traced to a myriad of mechanisms. For instance, the deposition of *S. haematobium* eggs on the bladder walls induces fibrosis with its attendant proliferation, hyperplasia, and metaplasia [96]. Again chronic urinary infection promotes the synthesis of nitrosamines from their urine precursors [97]. Therefore, it is not surprising that both the WHO and IARC have classified *S. haematobium* as a Class 1 carcinogen [9].

**Figure 1:**
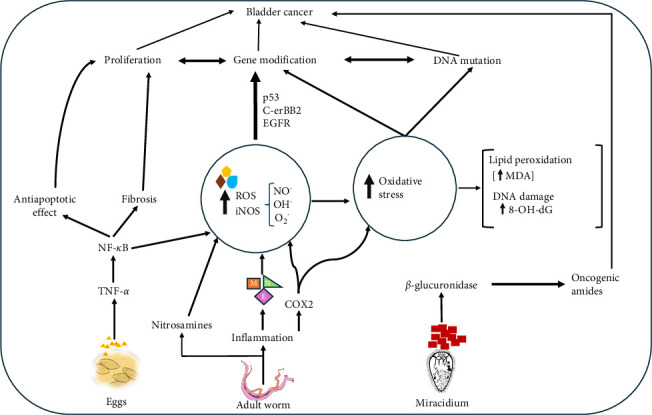
Proposed interplay between *Schistosoma haematobium* infection, oxidative stress, and the risk of bladder cancer.

Interestingly, nitrosamines modulate the production of nitrogen dioxide radicals, hydroxyl radicals, and superoxide anion radicals, thereby, inducing oxidative stress [98]. Further, evidence shows that during *S. haematobium* infection, both the miracidia and adult schistosomes influence the production of high levels of urinary *β*-glucuronidase with the subsequent release of carcinogenic amines in urine [99]. Schistosomiasis can also induce the overexpression of cyclooxygenase 2 [100], a known promoter of oncogenesis [101]. Finally, in *S. haematobium* associated squamous cell carcinoma, multiple oncogenic mutations and abnormal expression of certain vital genes and proteins (e.g., c-erbB-2, p53, and epidermal growth factor receptors) have been observed [102, 103]. An illustration of the interaction between *S. haematobium* infection, oxidative stress, and the risk of bladder cancer is shown in Figure 1.

*Schistosoma haematobium* eggs deposited in the bladder walls release antigens which promote TNF-*α* and NF-*κ*B release. Activated NF-*κ*B leads to iNOS-mediated increased NO, OH^.^ and O_2_^.^ radicals leading to oxidative stress. Adult worms cause inflammation and release nitrosamines, leading to further elevating ROS. Again, inflammation causes the accumulation of E, M, and N, which also contribute to iNOS and ROS production. Excessive ROS production leads to oxidative stress marked by increased lipid peroxidation (MDA, DNA, and protein oxidation (8-oxo-dG). This eventually influences gene mutation, DNA damage, proliferation, and cancer. Urinary glucuronidase produced by the miracidium and cyclooxygenase-2 (COX 2) generated through inflammation by adult worms could lead to the synthesis of oncogenic amides which could lead to cancer.

The data on *S. haematobium's* impact on carcinogenesis are the strongest, but it remains unclear if *S. mansoni* and other *S. japonicum* species possess a direct carcinogenic potential [104]. Available evidence shows that *S. mansoni* could generally act as a cofactor for a hepatic lesion in Hepatitis B and C virus infections, potentiating liver injury [105]. In *S. mansoni* infections, antigens released from schistosome eggs trapped in tissues influence the release of proto-oncogenes associated with hepatocellular carcinoma [106]. Among these are the transcriptional factors STAT3 and c-jun, both of which are crucial in the molecular pathway leading to inflammation and the development of cancer. Therefore, targeting these underlying pathways could be useful in providing therapeutics for schistosomiasis-related carcinogenesis [106].

## 5. Future Perspective and Conclusion

Schistosomiasis remains a public health burden, especially in less developed countries and the need to find a lasting solution is apparent. More worrying is the fact that some species such as *S. haematobium* is known to carry some risk for cancer development.

Various studies have established the importance of oxidative stress in schistosome infection. This is enabled through oxidative damage to DNA and lipid peroxidation and direct immunologic complications leading to fibrosis. There is a huge interplay between *S. haematobium* infection and bladder cancer mediated by oxidative stress. Thus, it is our opinion that treatment of these infections should be considered alongside the remediation and if possible, the reversal of the damages caused by the oxidative insults. In this review, we have elucidated that redox activity in the schistosomes is regulated based on its developmental stage and thus, the expression of antioxidant enzymes by the flukes is minimal in the schistosomula. Therefore, diagnosis and treatment of schistosomiasis in the early stages are crucial to avoid further complications. Therefore, drugs targeting the schistosomula may have maximum effect during the treatment of the infection in the early stages. Consequently, future studies should be directed at elucidating the mechanism needed to overcome the antioxidant systems of the parasite.

Comparatively, there is less data on the role ROS plays in modulating the molecular and metabolic activities involved in S. *haematobium* and *S. japonicum* infections compared to *S. mansoni*. Most attention has been given to oxidative DNA damage caused by *S. mansoni* species when compared to other human parasitic flukes such as *S. haematobium* and *S. japonicum*. Interestingly, it is *S. haematobium* that has been classified as a class I carcinogen by the WHO. It is, therefore, suggested that more studies focusing on the mechanisms connecting ROS with cancer development and progression should be carried out.

## Data Availability

All data generated or analyzed during this study are included in this published article.
